# Cost-Effectiveness between Double and Single Fecal Immunochemical Test(s) in a Mass Colorectal Cancer Screening

**DOI:** 10.1155/2016/6830713

**Published:** 2016-04-07

**Authors:** Shan-Rong Cai, Hong-Hong Zhu, Yan-Qin Huang, Qi-Long Li, Xin-Yuan Ma, Su-Zhan Zhang, Shu Zheng

**Affiliations:** ^1^Cancer Institute, Key Laboratory of Cancer Prevention and Intervention, China National Ministry of Education, Key Laboratory of Molecular Biology in Medical Science, The Second Affiliated Hospital, School of Medicine, Zhejiang University, Hangzhou, Zhejiang 310009, China; ^2^Preventive Medicine Institute, Louisiana, MO 63353, USA; ^3^Institute of Oncology Prevention and Treatment, Jiashan, Zhejiang 314100, China

## Abstract

This study investigated the cost-effectiveness between double and single Fecal Immunochemical Test(s) (FIT) in a mass CRC screening. A two-stage sequential screening was conducted. FIT was used as a primary screening test and recommended twice by an interval of one week at the first screening stage. We defined the first-time FIT as FIT1 and the second-time FIT as FIT2. If either FIT1 or FIT2 was positive (+), then a colonoscopy was recommended at the second stage. Costs were recorded and analyzed. A total of 24,419 participants completed either FIT1 or FIT2. The detection rate of advanced neoplasm was 19.2% among both FIT1+ and FIT2+, especially high among men with age ≥55 (27.4%). About 15.4% CRC, 18.9% advanced neoplasm, and 29.9% adenoma missed by FIT1 were detected by FIT2 alone. Average cost was $2,935 for double FITs and $2,121 for FIT1 to detect each CRC and $901 for double FITs and $680 for FIT1 to detect each advanced neoplasm. Double FITs are overall more cost-effective, having significantly higher positive and detection rates with an acceptable higher cost, than single FIT. Double FITs should be encouraged for the first screening in a mass CRC screening, especially in economically and medically underserved populations/areas/countries.

## 1. Introduction

Colorectal cancer (CRC) is a significant burden on global health [1]. CRC is a leading cause of cancer death worldwide and its incidence and mortality are increasing in China and Japan lately [2]. In China, the overall cumulative incidence of CRC is 28.1 per 100,000 ranking the third in cancer incidence spectrum, 32.3 per 100,000 in urban populations ranking the second, and 35.6 per 100,000 in Jiashan County ranking the first during 2003–2007 [3]. Mass CRC screening is confirmed to be effective in CRC control and prevention, showing a significant decrease of CRC mortality by 15–33% with fecal occult blood tests (FOBT) [4–8]. Many mass CRC screening protocols/strategies in the world have been reported [1, 2, 9]. FOBT, flexible sigmoidoscopy (FS), and colonoscopy are recommended by US for CRC screening [2, 10, 11]. However, to date, there is no consistently preferred protocol/strategy of mass CRC screening. Every method has pros and cons [2]. Considering the cost-effectiveness and given that the evidence to date does not suggest a significant difference in cost-effectiveness between the three primary screening tests (FOBT, FS, and colonoscopy) in CRC control and prevention [12, 13], noninvasive and inexpensive FOBT is still a better primary mass CRC screening test than FS and colonoscopy, especially for the economically and medically underserved populations, areas, and countries.

Among all FOBT methods, fecal immunochemical occult blood test (FIT) is recommended by the National Comprehensive Cancer Network, USA [http://www.nccn.org/], and widely accepted in mass CRC screenings. FITs have several improved features compared with the standard guaiac FOBT [2, 13–16]. They are not subject to interference from animal blood in the diet, and they are more sensitive for detection of CRC and advanced adenomas [13–17]. Reported sensitivities for FIT to detect colorectal neoplasm range from 40.5% to 67% [18–20] and our previous screening result based on natural community populations showed that 9.5% CRC cases were missed by one RPHA-FOBT [21]. Annual FOBT has been reported to reduce more mortality than biennial FOBT (33% versus 18–21%) [2–7]. Thus, to improve the sensitivity in the primary screening, repeated FITs are necessary but how much more cost-effective one additional FIT is than single FIT in a mass screening of CRC remains unclear. This study investigated double FITs of two stool samples collected at different time compared to a single FIT based on cost and effectiveness measures in a free mass CRC screening in a rural population in China.

## 2. Methods

### 2.1. Study Design and Population

Permanent residents in Jiashan County, China, were our source population. Our inclusion criteria included all permanent residents who were living in the three randomly selected communities in Jiashan County and aged 40 to 74 years in Gan-Yao in 2007, Da-Yun in 2008, and Yao-Zhuang in 2009. Based on the inclusion criteria, 31,963 permanent residents from these three communities were our study population. All these eligible residents were invited to attend a free CRC screening program. A total of 24,419 study participants signed the written informed consent and participated in this CRC screening program. Basic characteristics of the study participants and positive predictive value of this screening have been reported by Cai et al. [22].

A two-stage sequential screening was designed and conducted. FIT was used as a primary screening test at the first stage of this mass CRC screening. Two stool samples were collected with an interval of one week by community health workers and tested in a local hospital by contracted experienced technicians. Three different parts were taken from each stool sample and then mixed and washed by special buffer solution. Each sample was collected in a bottle, about 5 mL moist stool content. All samples were tested in the laboratory immediately after collection. The second sample was collected in one week after the first one. FIT test using colloidal gold assay (monoclonal antibody) could detect a level of human hemoglobin as low as 0.05 *μ*gHb/mL and be done in less than five minutes. FIT test kits were purchased from WHPM, Inc., in Beijing, China. In this study, we defined the first-time FIT as FIT1, the second-time FIT as FIT2, and either FIT1 or FIT2 as FITs. If any participants have either FIT1 or FIT2 positive (FITs+), then a colonoscopy was recommended at the second stage of screening. Polyethylene Glycol Electrolyte Powder was used as a preparation drug for colonoscopy. If a colonoscopy examination failed due to inadequate bowel preparation or the cecum could not be reached for some reason, a subsequent colonoscopy was performed within one month.

All of the above examinations including FITs and colonoscopy were free to participants. Histopathological examination of CRC, adenoma, and nonadenomatous polyps has been reported by Cai et al. [22]. This study has been reviewed and approved by the Ethics Committee at Zhejiang University Cancer Institute.

### 2.2. Statistical Analysis

SPSS 16.0 software was used to do data analysis. Positive rate was calculated as the number of positive FITs divided by FIT participants. Detection rate was calculated as the number of detected cases divided by colonoscopy participants. Positive and detection rates in percent, odds ratios (ORs), and 95% confidence intervals (CIs) and the costs in both Renminbi, CNY (¥), and US dollars ($) were estimated by FIT and colonoscopy, respectively. Chi-squared tests were used to test the differences in positive and detection rates, and ORs between FIT1, FIT2, and FITs. If more than 20% expected frequencies of the events were below 5 in fourfold (two-by-two frequency) tables, then Fisher's exact test was used and a rank test was used in multiple contingency tables. Advanced adenoma was defined as adenoma ≥10 mm, or with a histology showing either a ≥20% villous component or high grade dysplasia. Advanced neoplasm was defined as either CRC or advanced adenoma in the analyses.

### 2.3. Cost Estimation

The cost of FIT1 was CNY ¥8.00 (Renminbi) per case including ¥5.00 for the purchasement of test kits, ¥1.50 for sample collection, ¥0.50 for testing fee, and ¥1.00 for test organization. The cost of FIT2 was ¥7.00 per case because the fee for test organization has been done by FIT1. The total cost of colonoscopy was ¥270.00 per case. The currency exchange rate between CNY Yuan and US dollar was ¥6.357 for one USD ($1.00) on August 27, 2012. Other costs such as CRC treatment fees were paid by participants themselves.

## 3. Results

The overall compliance rate for FITs was 76.4%, with 24,419 participants completing at least one FIT (either FIT1 or FIT2) among the total study population (*N* = 31,963). The compliance rate was 76.3% (24,375/31,963) for FIT1 and 65.3% (20,886/31,963) for FIT2. A significant lower compliance rate was for FIT2 comparing to that for FIT1 (*P* < 0.01). The overall compliance rate for colonoscopy was 81.2% (1,430/1,762) among FITs+ participants [22], ranging from 80.7% to 83.1% in any combinations between FIT1 and FIT2.  Table 1 presents OR and 95% CI of positive FIT and compliance to colonoscopy by gender, age group, and FIT status in this mass CRC screening program. OR (95% CI) of FITs for the compliance to colonoscopy was 1.03 (0.86–1.25) using FIT1 and 0.92 (0.75–1.13) using FIT2 as reference.

The positive rate was 4.7% (1,148/24,375) by FIT1 and 4.4% (915/20,886) by FIT2. There was no statistical difference in the positive rate between by FIT1 and FIT2 (*P* > 0.05). The positive rate by either FIT1 or FIT2 was 7.2% significantly higher than that by FIT1 and FIT2, respectively, shown in Table 1. OR (95% CI) of the positive rate by either FIT1 or FIT2 was 1.57 (1.46–1.70) using FIT1 as a reference and 1.70 (1.56–1.84) using FIT2 as a reference. There was no statistical difference in positive rate by either FIT1 or FIT2 between men and women (7.1% (841/11,808) among men and 7.2% (914/12,611) among women). The positive rate by either FIT1 or FIT2 was significantly higher among participants with age ≥55 than that among those with age <55 in both genders (9.1% versus 5.5% among men and 8.0% versus 6.6% among women).

There were 39 CRCs and 211 adenomas (88 advanced adenomas and 123 nonadvanced adenomas) patients including 127 advanced neoplasms patients detected by colonoscopy among 1,430 participants with positive FITs. ORs and 95% CIs of detection of CRC and adenoma using colonoscopy as a gold standard by gender, age group, and FIT status are presented in Table 2. ORs of detection of CRC and adenoma by either FIT1 or FIT2 were not statistically significant in all groups whenever FIT1 or FIT2 was used as reference, respectively. But the detection rates of both CRC and adenoma by either FIT1 or FIT2 are shown to be significantly higher among participants with age ≥55 than that among those with age <55 in both genders (OR (95% CI) = 18.63 (2.50–139.05) among men and 3.31 (1.06–10.36) among women). There was no significant difference in detection rate of CRC by either FIT1 or FIT2 between men and women. But there was a significant difference in detection rate of adenoma by either FIT1 or FIT2 between men and women. Men had higher detection rates of adenoma in all groups than women. ORs of detection of CRC by both FIT1 and FIT2 were statistically significant in the subgroups of those colonoscopy participants who had both FIT1 and FIT2 positive results (OR = 2.10 (1.16–3.80) using FIT1 as reference and OR = 2.36 (1.26–4.43) using FIT2 as reference) and all men who had both FIT1 and FIT2 positive results (OR = 2.33 (1.03–5.28) using FIT2 as reference), but they were not significant in the subgroups of men with age <55 and age ≥55, women with age <55 and age ≥55, and all women.

Table 3 presents OR and 95% CI of detection of advanced neoplasm by gender, age group, and FIT status. The detection rate of advanced neoplasm by both FIT1 and FIT2 was 19.2%, significantly improved with an OR (95% CI) = 1.90 (1.30–2.76) using FIT1 as reference and 2.25 (1.51–3.35) using FIT2 as reference, so were the rates among all men (1.82 (1.13–2.96) using FIT1 as reference and 2.34 (1.41–3.96) using FIT2 as reference) and all women (1.90 (1.30–2.76) using FIT1 as reference and 2.25 (1.51–3.35) using FIT2 as reference). There was a significant difference in detection of advanced neoplasm by both FIT1 and FIT2 between age <55 and age ≥55 in women but not in men. The detection rate of advanced neoplasm by both FIT1 and FIT2 in men was significantly higher than that in women. In addition, the detection rate of advanced neoplasm was significantly higher among men with age ≥55, men with age ≥55, men with age <55, and women with age ≥55, respectively, than that among women with age <55 in all combinations of FIT1+ and FIT2+.

The detection rates of various colorectal neoplasm by different positive combinations of double FITs are presented in Table 4. There was no significant difference in compliance rate for colonoscopy between two groups with positive FIT1 and FIT2 (*P* > 0.05). Comparing to FIT1+, the detection rate of CRC by FITs+ was not significantly improved; the detection rate of advanced adenoma by FITs was marginally significantly improved; and the detection rates of nonadvanced adenoma and advanced neoplasm by FITs were significantly improved (OR (95% CI) = 1.58 (1.19–2.10) and 1.38 (1.07–1.80), resp.). The detection rates were increased about 38% for advanced neoplasm and 58% for nonadvanced adenoma (45% for colorectal neoplasm) by FITs compared to FIT1 alone. There were 61.5% (24/39) CRC cases detected at an early stage (T_1-2_N_0_M_0_). Additional 15.4% (6/39, and among 6, 66.7% (4/6) were detected at an early stage) CRC, 20.5% advanced adenoma, 35.8% nonadvanced adenoma, and 18.9% advanced neoplasm cases missed by FIT1 were detected by FIT2 alone.

Costs in both Chinese Renminbi, CNY (¥), and US dollar ($) and detected CRC and advanced neoplasm by comparing double FITs to single FIT are presented in Table 5. Costs for the detection of CRC and advanced neoplasm by double FITs based on ¥8.00 ($1.22) for FIT1 and ¥7.00 ($1.07) for FIT2 per participant, respectively, were increased about 33–38%, $2,935 for double FITs and $2,121 for FIT1 for CRC and $901 for double FITs and $680 for FIT1 for advanced neoplasm. FIT2 found additional 6 CRC and 24 advanced neoplasm cases with an acceptable average cost of $7,401 and $1,850, respectively.

## 4. Discussion

This study investigated the performance of double FITs comparing to single FIT in a mass CRC screening in a rural population in China. The major findings indicated that double FITs were overall more cost-effective than single FIT. The positive rate was improved by double FITs comparing to FTI1 or FIT2 alone. The compliance rate for colonoscopy between FIT1 and FIT2 was similar. The detection rate of advanced neoplasm by double FITs was significantly improved comparing to FIT1. Double FITs found 18% more CRC and 38% more colorectal advanced neoplasms than single FIT1. A total of 15.4% (6/39) CRC, 18.9% advanced neoplasm, and 29.9% adenoma (including 20.5% advanced adenoma and 35.8% nonadvanced adenoma) detected by FIT2 alone would have been missed if only FIT1 had been used in the primary screening. The cost for CRC and advanced neoplasm detected by double FITs was increased about 33–38% which is acceptable and inexpensive comparing to 30 (6 CRC and 24 advanced neoplasm cases) lives saved from dying of colorectal advanced neoplasm cases and hundreds of other colorectal lesions cases prevented from developing CRC detected by FIT2 alone.

Some people in the community feel inconvenient and uncomfortable to collect stool samples, but stool samples are easily accessible and transportable, involving no painful procedure for collection, and can be done in privacy at home. Studies show that serum biomarkers such as M2PK and carcinoembryonic antigen (CEA) and/or combinations of these biomarkers could be a promising primary screening test in mass CRC screening [1, 2] but larger prospective studies using study populations representing a screening population are needed to verify promising results [23]. Serum CEA alone may not be a good screening test and it may be a little bit more expensive than FIT though it has been widely used in the surveillance of patients following primary surgical resection of CRC [24, 25] but it may be possible to be used as one of primary screening tests combining with other biomarkers such as FIT in mass screenings. Due to our limited budget, no serum biomarkers were used as primary screening in this mass CRC screening.

Overall, FOBT is relatively easy, safe, inexpensive, and acceptable comparing to colonoscopy which is limited by high cost, low participation rate, and variation in performance according to the endoscopist and high risk of pain and other adverse side effects [1, 2, 26–28]. Therefore, FOBT is still the first choice as a primary screening test for mass screening in many countries [16], especially in economically and medically underserved populations and areas [2, 7, 9]. Among all FOBTs, FIT is the most popular and acceptable primary screening test for its low cost and accessibility and a high adherence and detection rate of colorectal neoplasm [15, 16, 29] in mass CRC screening.

Sobhani has reported that screening program using FITs with three samples collected from three different parts of one-time stool at the same time is cost-effective [30]. But one sample for three FITs cost more than double FITs and may miss colorectal lesions with intermittent bleeding. Based on our decades' experience of mass CRC screening programs, the second FIT in one week after the first one is much easier to complete than that in one or two year(s) with a relatively lower cost without additional cost for organization and encouragement fees when the first FIT is completed. In addition, FITs with two stool samples collected at different time increase the detection rate of bleeding and may help detect those tumors with intermittent bleeding that may be missed by single FIT. Few studies, however, have been done about the effect of the number of FITs in mass colorectal neoplasm screening. Our study showed that the positive rate of FITs was significantly higher among FITs than that among FIT1 or FIT2 alone. It indicates that double FITs could find more high-risk participants than single FIT in the first stage of our mass CRC screening which reduced missing colorectal lesions. But additional 34.8% (614/1,762) individuals were correspondingly required to complete colonoscopy when comparing double FITs to single FIT. Based on this, it can be predicted that, for every 100,000 individuals screened with double FITs, approximately additional 3,480 individuals will require a colonoscopy compared to single FIT and 311 colorectal advanced neoplasms will be found at the same detection rate of 8.9% (127/1,430). Comparing to saving 311 lives dying of colorectal cancer and many lives prevented from developing CRC, costs for additional 3,480 individuals to have a colonoscopy are inexpensive and acceptable. Furthermore, it is important and necessary for participants to continue getting at least one FIT annually or biennially in the future after the first screening in order to prevent and control new and recurrent colorectal neoplasm.

The detection rate of advanced neoplasm and nonadvanced adenoma was significantly improved by 38–58% in double FITs+ compared to that in FIT1+. The detection rates of CRC and advanced adenoma were not significantly improved maybe due to a small number of CRC and advanced adenoma cases being detected. If the mass screening is applied among a larger population, the detection rates of both CRC and advanced adenoma would be significantly improved.

In our study, a total of 61.5% (24/39) CRC cases were detected at an early stage (T_1-2_N_0_M_0_) and 88 colorectal advanced adenoma cases were detected in this screening protocol. Thus, 88.2% ( = (24 + 88)/127) colorectal advanced neoplasm cases detected by screening were at an early stage of colorectal lesions. That is to say, these cases' lives were saved from dying of CRC after they received timely treatment and are getting more benefits from this screening for the rest of their life. A total of 15.4% CRC, 18.9% advanced neoplasm, and 29.9% adenoma (including 20.5% advanced adenoma and 35.8% nonadvanced adenoma) detected by FIT2 would have been missed if only FIT1 screening had been used. Double FITs found 18% more CRC and 38% more colorectal advanced neoplasms than single FIT1. The cost for CRC and advanced neoplasm detected by double FITs was increased about 33–38% which is acceptable because a FIT screening cost is about CNY ¥8.00 per person in China, equivalently about US $1.22 per person. The average cost was $2,935 for double FITs and $2,121 for FIT1 for CRC and $901 for double FITs and $680 for FIT1 for 108 advanced neoplasm cases. A total of $7,401 and $1,850 was the cost for additional six CRC and 24 advanced neoplasm cases by FIT2 only. But our study design of two FITs by an interval of one week saves additional 14.3% costs from FIT2 which costed ¥7.00 per participant comparing to ¥8.00 for FIT1. It would save millions of dollars in mass screenings if this screening protocol is used in China. Therefore, we deduce that double FITs by an interval of one week at the first stage is more cost-effective than single FIT in mass colorectal screenings in China, especially in medically and economically underserved populations, areas, and countries.

This study has some strengths. This is a large mass screening in a rural population in China. The compliance rate is relatively high. Our study design of two FITs by an interval of one week at the first screening stage can (1) help detect some colorectal lesions with intermittent bleeding which one FIT and two or more FITs from one sample may miss and the second FIT in one or two year(s) may be too late to diagnose, (2) save additional costs such as screening organization fees, and (3) increase compliance to the second FIT in one week. The cost analysis is based on the actual spent dollars and a comparative analysis between double FITs and single FIT within this screening program. The future benefits from this screening have not been included. Overall, results from this screening are reliable and valid. There are some limitations in this study. Some nonbleeding colorectal lesions may be missed due to the default of FOBT. False negative and false positive rates are relatively high because FITs have a relatively low sensitivity and specificity FIT comparing to colonoscopy.

## 5. Conclusions

Double FITs are more cost-effective than single FIT in our mass CRC screening based on the evidence of having significantly higher positive and detection rates with an acceptable higher cost by double FITs than single FIT. Double FITs should be encouraged for the first screening in a mass CRC screening, especially in economically and medically underserved populations/areas/countries.

## Figures and Tables

**Table 1:** Odds ratio (OR) and 95% confidence interval (CI) of fecal immunochemical test (FIT) positive and compliance to colonoscopy by gender, age group, and FIT status in the Jiashan mass colorectal cancer screening program in China, 2007–2009.

Gender	Age	FIT	Participant	Positive	OR1 (95% CI)	OR2 (95% CI)	Colonoscopy	OR1 (95% CI)	OR2 (95% CI)
			*n*	*n* (%)			*n* (%)		
Men	Age <55	FIT1	6,435	219 (3.4)	1.00		182 (83.1)	1.00	
		FIT2	5,314	196 (3.7)	1.09 (0.89–1.32)	1.00	162 (82.7)	0.97 (0.58–1.62)	1.00
		Both	5,302	62 (1.2)	0.34 (0.25–0.45)	0.31 (0.23–0.41)	54 (87.1)	1.37 (0.60–3.12)	1.42 (1.62–3.23)
		Either	6,447	353 (5.5)	1.64 (1.38–1.95)	1.51 (1.27–1.81)	294 (83.3)	1.01 (0.65–1.59)	1.05 (0.66–1.66)
	Age ≥55	FIT1	5,352	327 (6.1)	1.00		245 (74.9)	1.00	
		FIT2	4,589	253 (5.5)	0.90 (0.76–1.07)	1.00	196 (77.5)	1.15 (0.78–1.70)	1.00
		Both	4,580	93 (2.0)	0.32 (0.25–0.40)	0.36 (0.28–0.45)	73 (78.5)	1.22 (0.70–2.13)	1.06 (0.60–1.89)
		Either	5,361	487 (9.1)	1.54 (1.33–1.78)	1.71 (1.46–2.00)	368 (75.6)	1.04 (0.75–1.43)	0.90 (0.63–1.29)

Women	Age <55	FIT1	6,921	297 (4.3)	1.00		257 (86.5)	1.00	
		FIT2	5,918	224 (3.8)	0.88 (0.74–1.05)	1.00	198 (88.4)	1.54 (0.87–2.72)	1.00
		Both	5,911	64 (1.1)	0.24 (0.19–0.32)	0.28 (0.21–0.37)	57 (89.1)	1.27 (0.54–2.97)	1.07 (0.44–2.59)
		Either	6,928	457 (6.6)	1.58 (1.36–1.83)	1.80 (1.52–2.12)	398 (87.1)	1.05 (0.68–1.62)	0.89 (0.54–1.45)
	Age ≥55	FIT1	5,667	304 (5.4)	1.00		237 (78.0)	1.00	
		FIT2	5,065	242 (4.8)	0.89 (0.74–1.05)	1.00	198 (81.8)	1.27 (0.83–1.95)	1.00
		Both	5,049	82 (1.6)	0.29 (0.23–0.37)	0.33 (0.26–0.42)	66 (80.5)	1.17 (0.63–2.15)	0.92 (0.49–1.73)
		Either	5,683	464 (8.0)	1.57 (1.35–1.82)	1.77 (1.51–2.08)	369 (79.5)	1.10 (0.77–1.56)	0.86 (0.58–1.28)

Men	All	FIT1	11,787	547 (4.6)	1.00		432 (79.0)	1.00	
		FIT2	9,903	449 (4.5)	0.98 (0.86–1.11)	1.00	358 (79.7)	1.05 (0.77–1.43)	1.00
		Both	9,882	155 (1.6)	0.33 (0.27–0.39)	0.34 (0.28–0.40)	127 (81.9)	1.21 (0.76–1.91)	1.15 (0.72–1.84)
		Either	11,808	841 (7.1)	1.58 (1.41–1.76)	1.62 (1.44–1.82)	663 (78.8)	0.99 (0.76–1.29)	0.95 (0.71–1.26)

Women	All	FIT1	12,588	600 (4.8)	1.0		494 (82.2)	1.0	
		FIT2	10,983	459 (4.2)	0.87 (0.77–0.99)	1.0	396 (85.0)	1.23 (0.88–1.70)	1.0
		Both	10,960	145 (1.3)	0.27 (0.22–0.32)	0.31 (0.26–0.37)	123 (84.2)	1.16 (0.71–1.89)	0.95 (0.57–1.58)
		Either	12,611	914 (7.2)	1.56 (1.40–1.74)	1.79 (1.60–2.01)	767 (83.3)	1.08 (0.82–1.42)	0.88 (0.65–2.20)

Total		FIT1	24,375	1,148 (4.7)	1.0		926 (80.7)	1.0	
		FIT2	20,886	915 (4.4)	0.93 (0.85–1.01)	1.0	754 (82.4)	1.12 (0.90–1.41)	1.0
		Both	20,842	301 (1.4)	0.30 (0.26–0.34)	0.32 (0.28–0.37)	250 (83.1)	1.18 (0.84–1.64)	1.05 (0.74–1.48)
		Either	24,419	1,762 (7.2)	1.57 (1.46–1.70)	1.70 (1.56–1.84)	1,430 (81.2)	1.03 (0.86–1.25)	0.92 (0.75–1.13)

FIT1, the first FIT; FIT2, the second FIT; OR1 using FIT1 as reference; and OR2 using FIT2 as reference.

**Table 2:** Odds ratio (OR) and 95% confidence interval (CI) of detection of colorectal cancer (CRC) and adenoma using colonoscopy as a gold standard by gender, age group, and fecal immunochemical test (FIT) status in the Jiashan mass CRC screening program in China, 2007–2009.

Gender/age	FIT	Colonoscopy participant	CRC	OR1 (95% CI)	OR2 (95% CI)	OR3 (95% CI)	Adenoma	OR1 (95% CI)	OR2 (95% CI)	OR3 (95% CI)
			*n* (%)				*n* (*n* ^*∗*^), %			
Men <55	FIT1	182	1 (100.0)	1.00		1.0	37 (22), 75.5	1.00		1.0
	FIT2	162	1 (100.0)	1.12 (0.07–18.12)	1.00	1.0	25 (14), 51.0	0.72 (0.41–1.25)	1.00	1.0
	Both	54	1 (100.0)	3.42 (0.21–55.53)	3.04 (0.19–49.42)	1.0	13 (10), 26.5	1.24 (0.60–2.56)	1.74 (0.82–3.70)	1.0
	Either	294	1 (100.0)	0.62 (0.38–9.94)	0.55 (0.03–8.84)	1.0	49 (26), 100.0	0.78 (0.49–1.26)	1.10 (0.65–1.85)	1.0

Men ≥55	FIT1	245	19 (86.4)	1.00		15.23 (2.02–114.75)^a^	65 (23), 72.2	1.00		1.42 (0.89–2.24)^a^
	FIT2	196	13 (59.1)	0.85 (0.41–1.76)	1.00	11.44 (1.48–88.40)^b^	48 (15), 53.3	0.90 (0.58–1.38)	1.00	2.10 (1.23–3.58)^b^
	Both	73	10 (45.5)	1.89 (0.84–4.27)	2.23 (0.93–5.35)	8.41 (1.04–67.87)^c^	23 (10), 25.6	1.27 (0.72–2.25)	1.42 (0.79–2.56)	1.45 (0.66–3.22)^c^
	Either	368	22 (100.0)	0.76 (0.40–1.43)	0.90 (0.44–1.82)	18.63 (2.50–139.05)^d^	90 (28), 100.0	0.90 (0.62–1.30)	1.00 (0.67–1.49)	1.62 (1.10–2.39)^d^

Women <55	FIT1	257	3 (75.0)	1.00		1.0	16 (8), 64.0	1.00		1.0
	FIT2	198	2 (50.0)	0.86 (0.14–5.22)	1.00	1.0	14 (5), 56.0	1.15 (0.55–2.41)	1.00	1.0
	Both	57	1 (25.0)	1.51 (0.15–14.81)	1.75 (0.16–19.66)	1.0	5 (3), 20.0	1.45 (0.51–4.13)	1.26 (0.44–3.67)	1.0
	Either	398	4 (100.0)	0.86 (0.19–3.87)	1.00 (0.18–5.48)	1.0	25 (10), 100.0	1.01 (0.53–1.93)	0.88 (0.45–1.74)	1.0

Women ≥55	FIT1	237	10 (83.3)	1.00		3.73 (1.01–13.72)^e^	30 (17), 63.8	1.00		2.18 (1.16–4.12)^e^
	FIT2	198	8 (66.7)	0.96 (0.37–2.47)	1.00	4.13 (0.87–19.68)^f^	30 (14), 63.8	1.23 (0.71–2.13)	1.00	2.35 (1.20–4.58)^f^
	Both	66	6 (50.0)	2.27 (0.79–6.50)	2.38 (0.79–7.12)	5.60 (0.65–47.98)^g^	13 (7), 27.7	1.69 (0.83–3.47)	1.37 (0.67–2.82)	2.55 (0.85–7.66)^g^
	Either	369	12 (100.0)	0.76 (0.32–1.80)	0.80 (0.32–1.99)	3.31 (1.06–10.36)^h^	47 (24), 100.0	1.01 (0.62–1.64)	0.82 (0.50–1.34)	2.18 (1.31–3.62)^h^

Men All age	FIT1	432	20 (87.0)	1.0		1.0	102 (45), 73.4	1.0		1.0
	FIT2	358	14 (60.1)	0.84 (0.42–1.69)	1.0	1.0	73 (29), 52.5	0.83 (0.59–1.16)	1.0	1.0
	Both	127	11 (47.8)	1.95 (0.91–4.19)	2.33 (1.03–5.28)	1.0	36 (20), 25.9	1.28 (0.82–2.00)	1.54 (0.97–2.46)	1.0
	Either	663	23 (100.0)	0.74 (0.40–1.37)	0.88 (0.45–1.74)	1.0	139 (54), 100.0	0.86 (0.64–1.15)	1.04 (0.75–1.42)	1.0

Women All age	FIT1	494	13 (81.3)	1.0		0.56 (0.27–1.13)^a^	46 (25), 63.9	1.0		0.33 (0.23–0.48)^a^
	FIT2	396	10 (62.5)	0.96 (0.42–2.21)	1.0	0.64 (0.28–1.45)^b^	44 (19), 61.1	1.22 (0.79–1.88)	1.0	0.49 (0.33–0.73)^b^
	Both	123	7 (43.8)	2.23 (0.87–5.72)	2.33 (0.87–6.26)	0.64 (0.24–1.70)^c^	18 (10), 25.0	1.67 (0.93–3.00)	1.37 (0.76–2.47)	0.43 (0.23–0.82)^c^
	Either	767	16 (100)	0.79 (0.38–1.65)	0.82 (0.37–1.83)	0.59 (0.31–1.13)^d^	72 (34), 100.0	1.01 (0.68–1.49)	0.83 (0.56–1.23)	0.39 (0.29–0.53)^d^

Total	FIT1	926	33 (84.6)	1.0		—	148 (70), 70.1	1.0		—
	FIT2	754	24 (61.5)	0.89 (0.52–1.52)	1.0	—	117 (48), 55.5	0.97 (0.74–1.26)	1.0	—
	Both	250	18 (46.2)	2.10 (1.16–3.80)	2.36 (1.26–4.43)	—	54 (30), 25.6	1.45 (1.02–2.05)	1.50 (1.05–2.15)	—
	Either	1,430	39 (100.0)	0.76 (0.47–1.22)	0.85 (0.51–1.43)	—	211 (88), 100.0	0.91 (0.72–1.14)	0.94 (0.74–1.21)	—

FIT1, the first FIT; FIT2, the second FIT; OR1 using FIT1 as reference and OR2 using FIT2 as reference; OR3^a^ using men with age <55 who completed FIT1 as reference, OR3^b^ using men with age <55 who completed FIT2 as reference, OR3^c^ using men with age <55 who completed both FIT1 and FIT2 as reference, OR3^d^ using men with age <55 who completed either FIT1 or FIT2 as reference, OR3^e^ using women with age <55 who completed FIT1 as reference, OR3^f^ using women with age <55 who completed FIT2 as reference, OR3^g^ using women with age <55 who completed both FIT1 and FIT2 as reference, and OR3^h^ using women with age <55 who completed either FIT1 or FIT2 as reference.

*n* ^*∗*^: the number of advanced adenomas.

**Table 3:** Odds ratio (OR) and 95% confidence interval (CI) of detection of advanced neoplasm using colonoscopy as a gold standard by gender, age group, and fecal immunochemical test (FIT) status in the Jiashan mass colorectal cancer screening program in China, 2007–2009.

Gender	Age	FIT	Colonoscopy participant	Advanced neoplasm	OR1 (95% CI)	OR2 (95% CI)	OR3 (95% CI )
				*n* (%)			
Men	<55	FIT1	182	23 (12.6)	1.0		1.0
		FIT2	162	15 (9.3)	0.71 (0.35–1.40)	1.0	1.0
		Both	54	11 (20.4)	1.77 (0.80–3.91)	2.51 (1.07–5.86)	1.0
		Either	294	27 (9.2)	0.70 (0.39–1.26)	0.99 (0.51–1.92)	1.0

Men	≥55	FIT1	245	42 (17.1)	1.0		1.43 (0.83–2.48)^a^
		FIT2	196	28 (14.3)	0.81 (0.48–1.36)	1.0	1.63 (0.84–3.18)^b^
		Both	73	20 (27.4)	1.82 (0.99–3.37)	2.26 (1.18–4.34)	1.48 (0.64–3.41)^c^
		Either	368	50 (13.6)	0.76 (0.49–1.19)	0.94 (0.57–1.55)	1.56 (0.95–2.55)^d^

Women	<55	FIT1	257	11 (4.3)	1.0		1.0
		FIT2	198	7 (3.5)	0.82 (0.31–2.15)	1.0	1.0
		Both	57	4 (7.0)	1.69 (0.52–5.51)	2.06 (0.59–7.30)	1.0
		Either	398	14 (3.5)	0.82 (0.36–1.83)	1.0 (0.40–2.51)	1.0

Women	≥55	FIT1	237	27 (11.4)	1.0		2.88 (1.39–5.94)^e^
		FIT2	198	22 (11.1)	0.97 (0.54–1.77)	1.0	3.41 (1.42–8.18)^f^
		Both	66	13 (19.7)	1.91 (0.92–3.95)	1.96 (0.93–4.16)	3.25 (1.00–10.61)^g^
		Either	369	36 (9.8)	0.84 (0.50–1.43)	0.87 (0.49–1.52)	2.97 (1.57–5.59)^h^

Men	All	FIT1	432	65 (15.0)	1.0		1.0
		FIT2	358	43 (12.0)	0.77 (0.51–1.17)	1.0	1.0
		Both	127	31 (24.4)	1.82 (1.13–2.96)	2.34 (1.41–3.96)	1.0
		Either	663	77 (11.6)	0.74 (0.52–1.06)	0.96 (0.65–1.43)	1.0

Women	All	FIT1	494	38 (7.7)	1.0		0.47 (0.31–0.72)^a^
		FIT2	396	29 (7.3)	0.95 (0.57–1.57)	1.0	0.58 (0.35–0.95)^b^
		Both	123	17 (11.4)	1.93 (1.05–3.54)	2.03 (1.07–3.84)	0.50 (0.26–0.95)^c^
		Either	767	56 (7.3)	0.95 (0.62–1.45)	1.00 (0.63–1.59)	0.60 (0.42–0.86)^d^

Both	All	FIT1	926	103 (11.1)	1.0		—
		FIT2	754	72 (9.5)	0.84 (0.61–1.16)	1.0	—
		Both	250	48 (19.2)	1.90 (1.30–2.76)	2.25 (1.51–3.35)	—
		Either	1,430	127 (8.9)	0.78 (0.59–1.02)	0.92 (0.68–1.25)	—

FIT1, the first FIT; FIT2, the second FIT; OR1 using FIT1 as reference and OR2 using FIT2 as reference; OR3^a^ using men with age <55 who completed FIT1 as reference, OR3^b^ using men with age <55 who completed FIT2 as reference, OR3^c^ using men with age <55 who completed both FIT1 and FIT2 as reference, OR3^d^ using men with age <55 who completed either FIT1 or FIT2 as reference, OR3^e^ using women with age <55 who completed FIT1 as reference, OR3^f^ using women with age <55 who completed FIT2 as reference, OR3^g^ using women with age <55 who completed both FIT1 and FIT2 as reference, and OR3^h^ using women with age <55 who completed either FIT1 or FIT2 as reference.

**Table 4:** Odds ratio (OR) and 95% confidence interval (CI) of detection of various colorectal neoplasm using colonoscopy as a gold standard by different positive combinations of double fecal immunochemical tests (FITs) in the Jiashan mass colorectal cancer (CRC) screening program in China, 2007–2009.

FIT1	FIT2	FITs	Positive	Colonoscopy	Results: *n*, detection rate, percentage^a^			
		*n*	*n*	*n*	CRC	Advanced adenoma	Nonadvanced adenoma	Advanced neoplasm
+	−	20,842	763	619	12, 1.9, 30.1	37, 6.0, 42.0	49, 7.9, 39.8	49, 7.9, 38.6
+	+	20,842	301	250	18, 7.2, 46.2	30, 12.0, 34.1	24, 9.6, 19.5	48, 19.2, 37.8
+	Absent	3,533	84	57	3, 5.3, 7.7	3, 5.3, 3.4	5, 8.8, 4.1	6, 7.1, 4.7
−	+	20,842	609	500	6, 1.2, 15.4	18, 3.6, 20.5	44, 8.8, 35.8	24, 4.8, 18.9
Absent	+	44	5	4	0	0	1, 25.0, 0.5	0
FIT1+^b^		24375	1,148	926	33, 3.6, 84.6	70, 7.6, 79.5	78, 8.4, 63.4	103, 11.1, 81.1
	FIT2+^c^	20886	915	754	24, 3.2, 61.5	48, 6.4, 54.5	69, 9.2, 56.1	72, 9.5, 56.7
Total		24,419	1,762	1,430	39, 2.7, 100.0	88, 6.2, 100.0	123, 8.6, 100.0	127, 8.9, 100.0
OR (95% CI) of detection rate by FIT (FIT2 versus FIT1)					0.85 (0.50–1.44)	0.80 (0.55–1.16)	1.03 (0.75–1.43)	0.82 (0.60–1.10)
OR (95% CI ) of detection rate by FIT (FITs versus FIT1)					1.18 (0.74–1.88)	1.26 (0.92–1.72)	1.58 (1.19–2.10)	1.38 (1.07–1.80)

FIT1, the first FIT; FIT2, the second FIT; FITs, combination of FIT1 and FIT2.

^a^Detection rate: the number of the colorectal lesions/the number of participants who completed colonoscopy in any combination of FIT1 and FIT2; percentage: the number of the colorectal lesions/the total number of the colorectal lesions in any combination of FIT1 and FIT2.

^b^Referred to those who completed FIT1 regardless of the completion of FIT2.

^c^Referred to those who completed FIT2 regardless of the completion of FIT1.

**Table 5:** Costs in both Chinese Renminbi, CNY (¥), and US dollar ($) and detected colorectal cancer (CRC) and advanced neoplasm by comparing double FITs to single FIT in the Jiashan mass CRC screening program in China, 2007–2009.

	FIT			Colonoscopy		CRC		Advanced neoplasm	
	Participant	Total cost (¥/$)^a^	Positive	Participant	Total cost (¥/$)^a^	Number	Cost (¥/$)^a^/case	Number	Cost (¥/$)^a^/case
FIT1	24,375	195,000/30,675	1,148	926	250,020/39,330	33	13,485/2,121	103	4,321/680
FIT2	20,886	146,202/22,999	915	754	203,580/32,025	24	14,574/2,293	72	4,858/764
Both	20,842	312,630/49,179	301	250	67,500/10,618	18	21,118/3,322	48	7,919/1,246
Either	24,419	341,510/53,722	1,762	1,430	386,100/60,736	39	18,657/2,935	127	5,729/901
FIT2 only	20,886	146,202/22,999	614	504	136,080/21,406	6	47,047/7,401	24	11,762/1,850

FIT1, the first FIT; FIT2, the second FIT; FIT2 only refers to those who completed FIT2 without the completion of FIT1.

^a^The currency exchange rate was 1.000 USD = 6.357 CNY at the time when the screening program was done.
